# Factors affecting paramedicine students’ learning about evidence‐based practice: a phenomenographic study

**DOI:** 10.1186/s12909-021-02490-5

**Published:** 2021-01-12

**Authors:** Anna Wilson, Susan Howitt, Adele Holloway, Anne-Marie Williams, Denise Higgins

**Affiliations:** 1 Faculty of Social Sciences, University of Stirling, FK9 4LA Stirling, UK; 2 Australian National University, Canberra, Australia; 3 University of Tasmania, Hobart, Australia

**Keywords:** Evidence‐based practice, Metacognition, Learning, Qualitative research

## Abstract

**Background:**

Evidence-based practice is an important component of pre-service professional learning in medicine and allied health degrees, including new programmes in paramedicine. Despite substantial interest in this area, there is still a lack of clear understanding of how the skills and understandings needed to develop the capacity to apply evidence-based practice can best be learned. Evidence-based practice is often described as consisting of five steps: ask, acquire, appraise, apply and assess. This study focuses on paramedicine students’ learning about the first three steps in a final year unit which explicitly aims to develop their skills in relation to these.

**Methods:**

We conducted a qualitative study of learning journals recorded by 101 of 121 students in a final year unit of a paramedicine degree (20 students either withheld consent for their journals to be used in the research or did not complete their journal entries). We used phenomenographic approaches to the data analysis in order to identify both variation in students’ learning and the factors affecting this variation.

**Results:**

We observed variation in students’ understanding of the purpose of literature analysis, the nature of medical research and its relationship to practice. In all three, we identify two main factors contributing to the variation in student learning outcomes: epistemological stance, and opportunities for metacognitive learning generated through peer interactions and self-reflection. We also found that as students begin to grapple with the complexity of medical research, this sometimes produced negative attitudes towards its value; such unintended outcomes need to be recognised and addressed.

**Conclusions:**

We suggest key factors that should be considered in developing coursework intended to enhance students’ understandings about the processes and application of evidence-based practice. Providing collaborative learning opportunities that address the architecture of variation we observed may be useful in overcoming epistemological and metacognitive barriers experienced by students.

## Background

Evidence-based practice (EBP) is crucial to all areas of health and medicine. Health professionals need to be able to access and evaluate clinical studies and then incorporate new findings into the treatment of patients [1], in a process often abbreviated as the five steps of ask, acquire, appraise, apply and assess [2]. That is, practitioners need to be able to develop appropriate questions, identify and evaluate relevant literature, apply what they have learnt to the clinical environment and then reflect on the outcome.

As EBP has become a defining feature of medical and allied health professional practice, it has also become a central part of pre-service professional education (see, e.g. [3–6]. Teaching and learning activities typically aim to include at least some of the five steps of EBP, often with a focus on the first three – ask, acquire and appraise – as these can be achieved in university-based, coursework settings [5–7]. Learning about these first three steps of EBP entails gaining an improved understanding of the nature of health and medical research, including processes, uncertainties and the presentation of results, as without such understanding, reliable evaluations of published studies cannot be made. As a result, research experiences of various types are increasingly being incorporated into medical and allied health degrees to assist students in recognizing, valuing and applying research [8, 9]. However, students enrolling in health and medicine degrees often choose such degrees because of their interest in patient care and outcomes [10]. This focus can inhibit them making links between research, where the relevance to practice may not be clear, and the basis for patient care. Such attitudes can form a significant barrier to successfully teaching EBP [5, 7].

The majority of existing literature addresses learning of EBP among medical students but it is just as important in the allied health professions [4, 6, 10, 11]. While existing evidence indicates that similar issues are emerging, it will be useful to understand any differences, which may have implications for developing teaching and learning activities, particularly as different health professions attract different student populations. Paramedicine is of particular interest because of its recent emergence as an academic discipline. In Australia and elsewhere, training of paramedics has moved, or is moving, from the vocational sector to becoming a full degree program. This is partly a response to changes within the profession itself, which now includes a wider diversity of activities with a greater role for professional judgment [12, 13]. Consequently, training now focusses more on critical thinking and EBP than on the adherence to protocols, which previously characterised the role [14]. In disciplines such as paramedicine that do not have a strong tradition of research, and where the relationship between a university education and professionalism may not be straightforward [15], a better understanding of student attitudes that may form barriers to acceptance of EBP is required.

EBP includes a complex set of activities, all of which must be mastered by students. Several systematic reviews identify learning gains from different approaches [3, 5, 6, 16] leading to the conclusion that there is no single best approach to EBP teaching and that it can be desirable to use multiple teaching strategies. Much of the focus of EBP teaching is on the earlier steps, using and analysing literature. While the later steps of application and assessment through reflection may require workplace settings for effective learning, the ask, acquire and appraise components may be more readily taught, and student learning more readily evaluated, in a university setting. Indeed, many studies have found that students develop skills in literature searching and the ability to critically analyse research through such approaches [3–7].

Many teaching strategies for EBP are underpinned by the assumption that once students engage with the literature, they will apply these skills in the professional environment. However, this is problematic because it requires not just the acquisition of knowledge and skills, but also changes in attitude and behaviours [3, 7, 11]. For EBP to be incorporated into professional practice, students must see their learning as more than the development of purely academic skills and instead as having clear links to practice. The current focus of much of the literature on the evaluation of interventions designed to teach EBP often fails to address how the student perceives the learning experience and how this affects learning outcomes. Similarly, the widespread use of quantitative evaluation methods results in average attainment being reported without an exploration of the nature of variation. Consequently, and despite the importance of student attitudes to EBP, there is a need for more work exploring how students understand and value the different components of EBP, which can then inform improvements to teaching and learning strategies [6].

In the present study, we have therefore chosen to adopt a qualitative approach that aims to explore the diversity of ways in which students experience and understand the first three steps of EBP. We present research into the understandings and attitudes students develop while engaging in an activity designed to introduce them to the ask, access and appraise steps of EBP and thus to enhance their understandings of EBP as a whole. We ask the following questions:

- What causes variation in observed learning outcomes in relation to EBP?
- Can students’ reflections in response to prompt questions be used to both reveal these causes and to assess their learning (and even to enhance their learning)?
- What are the implications for the design of effective strategies for teaching EBP?

Because our research questions focus on variation in observed learning outcomes, we draw on the methods of phenomenography [17–19]. Phenomenography seeks to describe the range of ways in which people can experience the same phenomenon, and in particular to identify the qualitatively different components or dimensions that are responsible for that range. The object of study of phenomenography is the “variation in … awareness or ways of experiencing a particular phenomenon” [17], and phenomenographic researchers aim to develop a set of descriptive categories that illustrate similarities and differences in how a particular phenomenon is or can be conceived of or experienced. In our context, phenomenography provides an approach to identifying and describing qualitatively different ways that students experience aspects of their learning about the role of EBP in paramedicine through their systematic literature review activity. The full range of variation is then encompassed in a set of categories, known as the outcome space. The categories that make up the outcome space are usually hierarchically related and inclusive, ranging from a relatively simple understanding to a complex and sophisticated view that takes multiple factors into account. Describing variation in this way can provide a framework for improved teaching strategies, as the different dimensions are effectively the critical components that need to be considered in designing activities for learning. We use this approach to identify three key dimensions to students’ developing understandings and attitudes about EBP that we suggest should be addressed when designing EBP-related learning activities.

## Methods

### Context and participants

Participants were Bachelor of Paramedic Practice students enrolled in a unit designed to teach EBP, Professional Development in Paramedic Practice. The unit is a capstone experience that is undertaken at the end of the degree program and aims to foster the integration of critical analysis and reflection into facets of professional practice. Students conduct a systematic literature review in groups and present their findings in several formats, including a conference-style presentation to both academics and staff from the local state ambulance service. These tasks account for most of the assessment within the unit. A minor assessment component is a reflective journal, described in the following section. It should be noted that the majority of students undertook a concurrent four-week emergency ambulance placement.

Table 1 provides demographic information on the cohort, who undertook the unit in 2016-7. The study had approval from the institution’s Social Science Human Research Ethics Committee, protocol number H0015518 to analyse students’ reflective journals. Informed consent was obtained from all individual participants included in the study. Of the 121 students in the unit, 20 either did not give permission for the journals to be included in the study or did not complete the journal, leaving 101 journals for analysis.

**Table 1:** Demographic characteristics of students enrolled in the unit (*n* = 121)

Demographic characteristics		% (number)
Gender	Female	52.9 (64)
	Male	47.1 (57)
Basis for admission	Year 12 leaver	47.9 (58)
	Other	52.1 (63)
Age	< 20	17.4 (21)
	20–29	66.1 (80)
	30–39	5.8 (7)
	40+	10.7 (13)

### Data collection and analysis

The students’ reflective journals formed the data set for this study. The reflective journal task was implemented in the university’s Learning Management System using a template that prompted students to reflect on their experiences during the systematic review task and to link them to practice. Students responded to questions about what they are doing and why, what problems they experience and what they are learning, at three points during the unit. Specific questions were designed to direct student attention to particular activities or learning outcomes. This approach was adapted from science research projects [20] and avoids issues of student concern about unstructured reflective writing and staff concern about assessing reflective writing, since marks are allocated for completing the task. Several questions were designed to prompt metacognition and self-reflection, which may contribute to more effective training as health professionals [21]. The question sets are provided in Table 2. All students responded to the same questions in the first and final set. In responding to the middle set, students could choose the questions most relevant to them and were expected to answer at least three.

**Table 2:** Question Bank for Reflective Journal

First Post Questions to be answered prior to research project / experience In your first post please respond to all four of these questions: 1. What are you expecting to get out of this research project / experience? 2. Have you undertaken a research project previously? If so, describe it. 3. What are you expecting to be different in this project experience from your normal course work? What skills do you think you need to be a good researcher?
**The Question Bank from which questions can be chosen during the research project / experience** In framing your second response you should answer any 3 questions. Here are the questions that we would like you to select from: • How have your recent activities helped you address your research question? • Have you made progress in the last fortnight? o If so, what allowed you to make progress? o What kind of activities did you engage in that helped you make progress? • Problems and obstacles are a normal part of research. Did you encounter any? o If so, what made them problems? o How did you go about solving them? o What would have helped you overcome them? • What might you have done differently if you had known two weeks ago what you know now? • Has your research question changed? If so, why, and what has it changed to? • Have you found/learned anything unexpected? Explain. • Has anything you’ve learned shifted the focus or aims of your project? How? • How confident are you in drawing any conclusions from your observations or results? Why? • How have you chosen the approach or methods that you are using for your project? • What are the connections between your research activities and your other studies? • Can you see ways in which you could apply what you have learned to other activities, in or out of university? How? • What have you learned about your project topic, science or research more generally? • What have you learned about yourself from doing this project? • Has your view of what research is changed from your project experience? Explain how.
**Last Post Questions to be answered at the end of the research project / experience** In your last post please respond to all four of these questions: 1. Has your research project/experience met your expectations? Why/why not? 2. What have you learned from undertaking this research project/ experience? 3. Would you do another research project / experience if you had the opportunity? Why/why not? 4. What skills do you think you developed or strengthened through your research project? What role do you think research skills will play in your future profession?

As can be seen from Table 2, the question sets were not intended as a survey tool designed to assess students’ level of knowledge about EBP, but rather to surface the ways in which they were experiencing their learning, and relationships between their experiences and apparent learning outcomes. As such, they are not a survey tool that requires conventional validation or reliability testing. However, it is important to note that the question sets used in this study were created by adapting similar question sets that have been successfully used to surface student experience and understanding in undergraduate science [20].

To explore students’ conceptions of different aspects of EBP, we conducted a qualitative analysis of student responses. All journals were de-identified and entered into NVivo to facilitate analysis of data and identification of material relevant to particular themes. Because we were interested in the different ways in which students understand EBP and how it is integrated with professional practice, we adopted a phenomenographic approach to the analysis [17, 18, 22]. We used this approach to identify different “dimensions” that describe the ways in which students experience and understand the first three steps of EBP as they engage in their literature review assignment, and qualitative variation within these dimensions.

Initial thematic analysis was conducted by SH, DH and AW, who have backgrounds in educational research but not paramedicine. This gave them an outsider’s perspective on both the unit and the discipline. Discussion of the initial themes identified different dimensions of experience and variation in student responses within each dimension. These categories were then used to construct an outcome space that described the nature of variation across the different dimensions. The outcome space was peer-validated by the other authors (AH and A-MW), who were familiar with the unit, degree structure and discipline, thereby providing insider knowledge. Analysis proceeded iteratively, following an approach similar to those described by Åkerlind (2012). That is, the students’ reflective journals were treated as a single pool of data, read and re-read in their entirety. Phenomenography recognises that students may exhibit different levels of sophistication in response to different prompts or contexts and at different times, and thus explicitly avoids categorising or labelling individual students. Instead, categories are developed by treating the data as a whole as a representation of the range of possible experiences and understandings. This process allowed us to identify the main *dimensions of experience* [18], that is, dimensions or facets of the activity that students held varying conceptions about and that thus limited or opened up what they were able to learn. Initial themes and categories were developed by AW, SH and DH, and discussed and refined until all five authors were in agreement. The final step in the analysis was then to seek explanations for the variation observed within these dimensions, relating lower and higher levels of sophistication to factors including opportunities for social learning and students’ epistemological stance, as described below in the discussion.

## Results

The students’ responses to the prompt questions in their journals revealed significant variation in both how students experienced the literature research activity and the learning that resulted from it. Our analysis suggests that these experiences and learning outcomes depended on three main dimensions of experience. The main purpose of the literature review activity was to introduce students to the processes needed to frame and answer a question by searching literature – the ask, acquire and appraise steps of EBP. The students’ comments revealed substantial variation in their understanding of this process. In addition, the comments showed two other important dimensions that contributed to the students’ overall learning: conceptions of the production and nature of medical research knowledge, and conceptions of the ways in which medical research knowledge integrates with practice.

In the following, we first describe the results of the phenomenographic analysis, outlining the three key dimensions of experience that emerged, and describing the qualitative variation within these dimensions and relationships between them. We then provide evidence from the reflections that suggests that social learning opportunities may be particularly important if the more sophisticated levels are to be achieved.

### Phenomenographic outcome space

The phenomenographic outcome space that captures this variation is shown in Table 3. As is often the case in phenomenographic analyses, the qualitative variation in each dimension takes the form of a nested hierarchy, across which conceptions expand and evolve. As will be discussed in the next section, the variation in each dimension is structured in similar ways, mapping both to the different levels of sophistication described by the Structure of Observed Learning Outcomes (SOLO) taxonomy [23] and the different epistemological stances identified by Perry [24]. We provide more detail on each dimension in the following subsections.

**Table 3:** Dimensions and variation in students’ experiences and conceptions of learning about EBP in the literature research activity

Dimension of experience	Category 1 (least sophisticated)	Category 2	Category 3	Category 4 (most sophisticated)
A. Processes needed to frame and answer a question by searching literature	No evidence of learning processes for literature analysis, focus on answer to question or use of search techniques	Acquires and applies search and limited evaluation strategies and believes this is sufficient to answer a question	Recognises complexity but may be confused or feel that no reliable judgements can be made because the literature is not trustworthy	Recognises the need for critical analysis in judging which research findings to believe
B. Production and nature of medical research knowledge	Expects a clear answer, frustrated if not acquired – no evidence of learning about the nature of research	Recognises differences in quality of research and explains with reference to bias or poor practice	Recognises differences in reliability of research and limitations of research with human subjects but has difficulty in evaluating such issues	Recognises uncertainty as inherent to medical knowledge, and knowledge as dynamic and therefore needing continual production
C. Ways in which medical research knowledge is integrated with practice	Sees no relevance to practice	Sees research as relevant to practice but sees integration as largely unproblematic	Focus on tension between scientific and practice knowledge but without ability to resolve	Recognises need to critically combine scientific knowledge with clinical experience (own and others) and patient’s unique context

#### Dimension A: processes needed to frame and answer a question by searching literature

As noted above, the primary aim of the literature research activity was to provide students with the opportunity to develop their skills and understanding in relation to the first three steps of EBP – ask, acquire and appraise. During the unit, the students were introduced to the mechanics of searching online databases, and most students did report gains in this ‘acquire’ part of the process. However, the students’ responses to the prompt questions revealed a significant range in the ways they understood the wider processes needed to ‘ask’ and ‘analyse’, even by the end of the semester.

Indeed, a small number of students showed no evidence of having engaged in or understood the importance of these elements of the process. This was evident in responses that were limited entirely to mastery of search engines and databases, such as “I learnt the efficiency of search engines and how to navigate through them with ease” (ID79) and “I have learned extensively about the topic … [and] how to expand my research strategies in a number of different databases” (ID83). Responses in this category are represented by the left-most cell in the first row of Table 3.

However, most students did develop a more sophisticated understanding of the other steps that contribute to the overall process of framing and answering a question using the medical research literature, in particular the ‘appraise’ step. Some students demonstrated that they had acquired (limited) evaluation strategies in addition to search techniques, as illustrated by ID98’s comment, “I have learned the correct way to research a topic and now know factors that make a study valid/bias” and ID77’s comment, “I have learnt how to properly interpret research results.” Both these comments suggest that the students have recognised the need to differentiate between different studies, but also that they see such differentiation as unproblematic once the right techniques have been mastered.

Increasing sophistication was evident in the responses of those students who had indeed recognised that analysing the literature is not an easy or straightforward process. This, however, led to two possible conceptions of the ‘appraise’ step of EBP – either that this complexity was irresoluble, often because the literature is not trustworthy, or that judgements about research require critical analysis (and that this is an ability that develops with time). The former category was evident in comments such as the following:

I am not very confident in drawing conclusions because all of the research we found is generally of poor quality. There are no randomised control trials or double blinded studies. (ID11)

Examples of the latter category, represented by the right-most cell of the first row in Table 3, include the following: “I have learnt that analysing literature is a complex skill that needs to be developed with much practise and guidance” (ID101). Occasionally, responses in this category would explicitly reference the student’s recognition of how their conceptions of the processes needed to ask, access and appraise had changed during the module, as illustrated in the following:

I have learnt that many of my searches in the past were quite biased as I would research in a way to find evidence for what I was thinking or what I wanted to hear, rather than assessing all available evidence. This caused me to often prove my pre-conceived judgements on a topic, rather than to question myself and possibly change my views based on evidence available. Furthermore, I would often believe information presented in a report or results obtained from an experiment without reviewing the methods used or validity of evidence provided. (ID107)

#### Dimension B: production and nature of medical research knowledge

While developing students’ understanding of the nature of medical research knowledge and the ways in which it is produced is not an explicitly-framed learning outcome for the unit, it became clear from the comments in the students’ journals that this was an important dimension that contributed to their learning about EBP. As in the first dimension of experience described above, these comments revealed increasingly sophisticated conceptions that are represented by the second row in Table 3.

The least sophisticated conceptions, on the left-most side of the table, suggested that, for some students, engaging with the research literature had not dislodged a rather black-and-white conception of medical research knowledge. Students with this conception were focused on obtaining a clear, correct answer, and showed no evidence of learning about variation, ambiguity and uncertainty in research. This attitude was evident in comments such as the following, which shows how the frustration caused by failing to find a perceived clear answer was often associated with frustration and disappointment with the unit: “no, we did not obtain a clear answer to our question. i feel disheartened that the amount of effort the group put into this work and only to receive an unclear answer was disappointing” (ID21).

Some students did explicitly refer to differences in research quality, but were only able to explain this with reference to bias or poor practice. Comments illustrating this category included the following: “I’m amazed at how much statistics can be manipulated or ignored” (ID58), “[I have learned that] many studies are poorly planned and have small sample sizes, resulting in a lack of statistically significant findings at completion” (ID62) and “[r]esearch often seems to be poorly gathered and without control groups. Much bias exists with medical studies” (ID36). This unintentional undermining of students’ confidence in medical research was particularly clear in the following excerpt: 

… this project has made me more cynical about research. Learning about bias and where it comes from has reduced my faith in evidence based practice. Other things like blatant conflict of interests for authors, individuals from study populations going “missing” and several other things. (ID27)

Other students integrated a recognition of variability in the reliability of research with an understanding of the limitations of doing research with human subjects. However, as with the two most sophisticated categories in the first dimension of experience described above, this additional understanding could result in two different conceptions. For some students, the realisation that medical research knowledge and knowledge production practices are neither black-and-white nor infallible left them feeling that judgements could not be made. Such a position is evident in the following comments:

… finding research that was as homogenous as we would have liked proved impossible. While each paper considered the difference between mechanical and manual chest compression, there were a lot of variables such as rate, depth, clinical setting, different devices used, differences in techniques and training, and different margins defined for success, that made drawing overall conclusions hard to answer our research question. (ID74)

… conducting research in paramedicine is a field fraught with ethical issues. There will always be limitations regarding what research can be conducted due to the potential outcomes (complications or death) of the patient in order to conduct a randomised controlled trial. (ID15)

In the first of these, ID74 recognises the inherent variability in research involving different mechanisms and taking place in different clinical settings. In the second, ID15 recognises that research involving humans experiencing the kinds of situation a paramedic might encounter in practice is inevitably fraught with ethical issues. In both cases, however, they have been unable to come to a clear conclusion about which of the research sources they have engaged with is more reliable, or to identify ways in which to resolve such issues of variability.

The final category associated with this dimension of experience differs from those illustrated with the preceding quotes in that the students’ recognition of the uncertainty in medical research is viewed much more positively, with some confidence that uncertainty can be examined and understood. ID56 demonstrates a mature understanding of research as a human endeavour, coupled with confidence that he/she has gained the ability to explain inconsistencies.

The project has perhaps given me some empathy to the challenges involved in conducting research, and helped to explain what seemed to be inconsistencies within research that I have seen previously. (ID56)

Some extended this view to recognise the value of engaging in continual knowledge production. The following excerpt shows how ID69 not only broadened his/her technical knowledge but also came to understand the importance of ongoing research as a knowledge-generating process:

Doing research on the topic has broadened my knowledge on weight calculations, paediatric drug dosages and drug preparations. I have also learnt different formulas for calculating different drugs for different aged children of different weights … I noted … the varying techniques/formulas/conclusions among studies from different countries. I have learnt that medicine is a research driven science and this entire unit has exemplified how important research is. (ID69)

#### Dimension C: Ways in which medical research knowledge is integrated with practice

The final dimension of experience we identified as shaping the overall learning of students in the unit is based on their conception of the relationship between medical research and practice knowledge, and in particular how research knowledge is integrated into practice. This was an area in which the learning evident in the students’ journals was somewhat disappointing; despite the inclusion of a prompt question that explicitly asked how relevant the unit would be to paramedic practice, only 40 % actually linked research to practice, with the rest seeing the systematic review as a purely academic exercise or commenting on generic skills such as teamwork. However, as with the two other dimensions, we observed a range of conceptions characterised by an expanding understanding and thus increased sophistication.

The simplest conception, represented by the left-most cell in the bottom row of Table 3, was one in which students essentially saw no connection between medical research knowledge and paramedical practice. This conception was evident in comments such as: “I don’t think these research skills will be relevant to my career” (D46) and “Directly to the profession, I do not see anything that we can take into our field of paramedicine, however for those who wish to assist or undertake studies and research within the specialty of paramedicine, there are many skills that can be taken into this, from the basic setting up of the topic and search parameters to the collation of data and analysis of this” (ID50).

Other students showed that they had recognised a connection between medical research and paramedical practice. However, there was again substantial variation in the ways this relationship was conceived. For some students, the connection was seen as largely unproblematic, with a linear and straightforward process of knowledge translation. Such a conception is evident in the following comments: “new literature is constantly being produced and to ensure best practice I need to keep up to date with the best information available” (ID5), in which the student suggests that all that is needed is to keep oneself informed.

Other students saw the relationship as problematic, but largely because they saw medical research knowledge as in tension or conflict with practice knowledge and lacked the ability to resolve this tension. This conception is evident in comments that recognised practice as having a limited foundation in knowledge generated through medical research, such as “a lot of our common practices only have limited evidence based support” (ID80). This particular example shows a student who has seen the tension and been led to doubt practice knowledge, but other students (particularly those focusing on bias and poor research practices in the second dimension above) were as likely to doubt medical research knowledge.

The most sophisticated conceptions of the relationship between medical research knowledge and paramedical practice knowledge, represented by the right-most cell in the bottom row of Table 3, showed students recognising the need to *critically* combine medical research knowledge with clinical experience. The following excerpt provides a clear illustration of this conception:

I have recently been on placement, having to think about the assignment I have asked current practicing paramedics about their thoughts on the research and their experience with the drug we have chosen to study. Hearing opinions from people currently practicing helps you consider different perspectives and also gives you insight that I may not have had from just reading the literature. ID51.

This was one of the few responses that linked placement experience (which most students were undertaking in parallel) with the systematic review.

In the most sophisticated instances, the patient’s unique context was also taken into account:

I think that a good researcher needs to be able to draw links, identify differences and notice trends throughout a large and varied body of information. I think that these skills are highly important for a paramedic. In order to understand a patient’s presentation a paramedic needs to be able to gather, compile and synthesise information to guide their decisions and patient treatment.‘ (ID91).

In many cases, the conceptions of each of the three dimensions expressed in the students’ responses to the prompt questions appeared to correlate with each other. That is, we observed several examples where the less sophisticated conceptions of the ask-access-appraise processes presented in the first row of Table 3 were expressed alongside less sophisticated conceptions of the nature and production of medical research knowledge and/or the relationship between medical research knowledge and practice knowledge.

Similarly, more sophisticated conceptions were often expressed simultaneously for each of the dimensions. Indeed, sophisticated conceptions of each dimension seemed often to occur as a result of the recognition of links between the dimensions, and thus as a result of deeper reflection and metacognition.

However, it is important to note that different levels of sophistication were also co-expressed, indicating that achieving rich, complex learning about EBP depends on the simultaneous development of each of the three dimensions. For example, some students appeared to hold quite sophisticated understandings of the processes needed to analyse the research literature while still being relatively unable to see how this was relevant to or could be applied within practice.

### Evidence for the value of social learning opportunities

The more sophisticated conceptions represented by the right-hand columns in Table 3 were sometimes explicitly described by students as having arisen as a result of peer interactions. Because the systematic review was conducted in groups, students had the opportunity to discover that their peers had different interpretations of the same findings. It appeared that this sometimes led students to see that simply finding a relevant research paper did not necessarily resolve a question. Those students who recognized the need for critical judgment and valued EBP often did so because they observed different responses to research from their peers or linked unit activities with their experiences in placement in ways that provided them with a contextual understanding of research.

The opportunity to reflect on how their own conceptions were evolving appeared to be an important addition to peer interactions. For example, for students such as ID48 and ID77, engagement with the ideas and interpretations of peers led to recognition of the ambiguities that are sometimes inherent in research, and thus to a deeper understanding of the need for professional judgment as a part of EBP:

ID48: … having several different members in the research group working on the same project has made me really appreciate the different ways in which other people view, gather and interpret research. I had not previously thought that practical research information would become so animated by different views, beliefs and opinions.

ID77: I have also learned that different people interpret studies differently as well, which surprised me, as people in my group, although reading the same studies had slightly different learning experiences from these studies.

A small number of students also indicated that they had found the social learning opportunities provided through their concurrent placement important in the development of their (more sophisticated) understandings. The excerpt from ID51’s journal given to illustrate Dimension C in the previous section is a good example of how exposure to the opinions of professionals in the practice context could further enrich students’ learning about their literature research topic. Another example is shown below, where ID78’s understanding of research is linked to his/her observation of variation in paramedics’ practices.

ID78: Being on placement, and being able to see our research question in practice has helped me address it, as it has helped give a more practical view of our question. Being able to see how an ambulance service prevents errors and how each individual paramedic has their own system and tools has helped my understanding of our research question.

Thus the use of prompt questions to facilitate reflection and metacognition seemed to be enhanced when coupled with opportunities for social learning.

## Discussion

Our analysis of students’ journals during an EBP unit has provided rich data on variation in paramedicine students’ attitudes towards both medical research knowledge and EBP. We have confirmed findings with both medicine and allied health students [3, 4, 6, 7, 10, 16, 25] that show students fairly readily gain skills in literature searching and some degree of analytical ability – the knowledge and skills components of EBP. To apply this learning to practice, however, requires changes in attitudes and behaviours. These are more challenging to change and maybe even more so with paramedicine students because of the recent emergence of paramedicine as an academic discipline. Tensions exist within paramedicine relating to its status as a professional discipline [15] and students’ attitudes to research may have been coloured by their views of the profession, particularly as the practical skills they learn are more clearly related to employment. Nevertheless, the attitudes we observed are similar to those seen in other health and medicine disciplines. We have extended previous studies by identifying considerable variation in student attitudes to learning and to professional practice, confirming findings with physiotherapy students [26]. We suggest that understanding the nature of this variation is a first step in promoting desired attitude changes. In the following sections, we explain the variation in terms of students’ epistemologies and the breadth of resources and perspectives they draw into the construction of their conceptions. We use this to suggest strategies to address students’ attitudes.

### Structure of variation in students learning about the ask, acquire and appraise steps of EBP: the influence of epistemological stance

The three dimensions we have described above share a common structure to the variation in sophistication within them (Table 3). First, the learning we are able to observe through the students’ comments may be mapped to the first four levels of the SOLO taxonomy [23] that is, prestructural, unistructural, multistructural and relational. The conceptions on the left-hand side of Table 3 can all be described as failures to grasp key aspects of the object of understanding or experience in question. Students holding these conceptions approach the literature research project as an exercise in acquiring information or a set of technical skills but do not engage with the broader structure in which those skills are exercised – that is, EBP. Conceptions in the second column suggest unistructural learning outcomes, in which students have identified one key aspect, such as bias or poor practice in medical research knowledge generation practices, but fail to move beyond that. The conceptions in the third column, which were often associated with feelings of frustration for the students holding them, appear to be multistructural in that students are aware of conflicting explanations or forms of knowledge but remain unable to resolve those conflicts and tensions by creating a relationship between them. The conceptions in the right-most column of Table 3 suggest that students who hold them have, indeed, been able to construct relationships between competing medical research evidence (Dimensions A and B) and knowledge generated through different means (Dimension C).

This common structure of variation in observed learning outcomes may be a result of different epistemological stances. Different epistemological stances (or conceptions of knowledge) are often described using a scheme drawn from developmental theories based on early work by Perry (1970) and further developed by several other researchers [27]. Setting to one side the considerable argument about whether the stances in the scheme are in fact developmental [28], it does provide a structure that can be used to evaluate students’ conceptions of different forms of knowledge, such as those derived from medical research or professional practice. In this scheme, the least sophisticated position is dualism, in which the student has an absolutist view and sees knowledge as right or wrong. Learning is therefore seen as the acquisition of facts. A more sophisticated stance is referred to as multiplicity, in which it is recognized that multiple viewpoints exist, but where students experience difficulty evaluating different views and may become confused or frustrated. Their learning may thus be directed towards seeking the views of an authority in order to resolve their confusion. A more sophisticated stance still is described as contextual relativism, in which students recognise the role of evidence and develop the ability to make judgements about different views based on the evidence. The most sophisticated stance in the scheme is one in which students not only make judgements, but are also able to commit to their own values, a stance often referred to as commitment within relativism.

The conceptions represented in the two columns on the left-hand side of Table 3 appear to be associated with a dualist stance, with students showing a tendency towards right/wrong and scientific/practice knowledge binaries. The conceptions associated with the third column appear to be associated with multiplicity, with students recognising conflicting versions of knowledge but unable to integrate or resolve them. These conceptions often resulted in students becoming frustrated or cynical about the value of research. The conceptions associated with the fourth column appear to be associated with a contextual relativist stance, with students feeling able to come to some critical judgement about the relationships between competing versions of truth or different forms of knowledge. These differences can also be considered in terms of coming to terms with uncertainty, which underpins the development of clinical judgement [29].

Because effective EBP requires students (and practitioners) to critically analyse and integrate the medical literature and the context of practice, it presupposes a more sophisticated epistemological state: students need to accept uncertainty to understand the contestability of knowledge [29]. Our study suggests that such sophistication cannot be presumed, in line with previous work showing that students in health and medicine degrees have relatively unsophisticated views of knowledge with only limited development during their degree in some cases (reviewed in [30]. This review also observed that most studies of students’ reactions to uncertainty did not explicitly consider epistemological foundations for their findings [30], implying that its importance may often be overlooked. Our findings thus lend further weight to calls for efforts to support students in developing more sophisticated epistemologies.

#### The impact of social learning and opportunities for reflection

The development of more sophisticated conceptions also seemed to depend on a students’ ability to engage with an expanding repertoire of sources from which to learn. The conceptions described in the left-hand side of Table 3 generally referred to learning from search engines and the literature, and/or from instruction on how to use search engines and databases. These students did not *reflect* on the processes of learning they were engaging in, except to identify them as frustrating when no clear answer was achieved.

The use of questions to prompt reflection did not, then, always result in the kind of reflection that might lead to the more sophisticated learning represented by the right-hand columns in Table 3. As described above, our data suggest that these more sophisticated outcomes were sometimes evident in reflections that referred to social learning, either through interactions with peers in the process of the group literature research, or through interactions with practising professionals in the parallel placement experience. That is, the most sophisticated understandings were achieved when students were able to engage in social learning processes and *also* to use reflective and metacognitive skills to identify their own learning through exposure to multiple perspectives.

Our findings thus support a proposal to include more collaborative learning opportunities within EBP teaching to expose students to alternative viewpoints [6]. However, many students did not demonstrate benefits from their group learning experience, supporting calls for more attention to process when designing group activities [31], particularly in the context of complex learning. Similarly, discussion with professionals is not always helpful, with other studies identifying clinical educators as a barrier to effective learning of EBP because they may not value it themselves [7, 25]. Strategies to address these issues must, therefore, take into account variation in student attitudes and in the professional environment [26].

### Implications for the design of learning activities aimed at increasing students’ understandings of the ask, acquire, appraise steps of EBP

Our analysis has established three key dimensions in the structure of students’ learning about the ask, acquire and appraise steps of EBP. It has also identified a clear structure within those dimensions that links learning outcomes from undertaking a literature research project to epistemological position, which can explain why some students achieved quite sophisticated understandings of the three dimensions, but others did not. Our findings suggest that opportunities for students to develop more sophisticated and integrated understandings can be increased if teaching and learning activities are designed to (i) explicitly address the three dimensions we have identified (that is, if the dimensions are used to “build a relevance structure” [19]; p143)); and (ii) expose students to the variation across each dimension (exploit the “architecture of variation “ [19]; p143)).

As described above, the journals show that some students failed to see the importance of learning a *process* for conducting literature research, rather than simply gathering information. Many more became disillusioned with research after learning about bias and poor practices. These were elements of the literature research activity that, left unaddressed, effectively hindered their learning and their ability to appreciate the nature and role of EBP in paramedic practice. By contrast, some of the more sophisticated responses were associated with enjoyment of the literature research process, leading to confidence in understanding and applying research. Early identification of those students who are engaging with the literature research in less sophisticated ways could be achieved through real-time monitoring of the students’ journals by tutors, followed by intervention. Such real-time targeted feedback to medical students struggling in clinical learning environments has been hypothesised to improve metacognitive and reflective abilities [21]. This suggests that unit learning outcomes would likely improve through feedback aimed at prompting deeper reflection and critical analysis of learning.

Our analysis also indicates that one of the main barriers to learning is students’ epistemological positions. We therefore suggest that activities need to be built into the learning process that allow students to recognise their own epistemological stance, become aware that others (students and professionals) may hold difference stances, and use this awareness to challenge their own positions and understandings. This would also need to take into account variation in students’ capacity for self-regulated learning and metacognitive awareness which is affected by age, personality and prior education [32]. Paramedicine students are drawn from a wide range of educational backgrounds [33] and the unit studied here included both students straight from school and more mature students with work experience or degrees in other areas (Table 1). A greater awareness of students’ initial attitudes [6] may inform the design of activities that promote epistemological development in this context. One possibility for including the social dimension, that has been trialled with paramedicine students, is student-tutor consensus assessment, where students can calibrate their reflections on practice through interaction with peers and tutors [34].

A further possibility for addressing this would be to adapt the questions and reflective journal used in the current work to include questions designed to prompt reflection and metacognition in relation to the dimensions and the conceptions of knowledge that underpin them. However, our analysis shows that it is often through discussions with others – peers and professionals in the placement context – that students are able to develop and express a position of contextual relativism. Individual reflections in journal entries may not provide sufficient opportunities for the exposure to other perspectives and interpretations needed to develop such a position, and we therefore suggest that more social learning opportunities that target underlying epistemologies are also needed. For example, students could be advised to discuss their project with paramedics and other professionals on placement, with questions specifically asking them to link their review and their placement experience.

### Limitations and further research

Our study relies solely on self-report, which could be seen as a limitation. However, this is an appropriate approach to address our interest in student attitudes. It could be complemented by observations of student skills and behaviour to confirm the perceptions recorded in the journals. In particular, while students wrote about their attitudes to professional practice, we have not observed whether these attitudes translate to practice. It would be interesting to determine if the more sophisticated journal responses do correlate to the use of EBP in a professional environment, which could further validate the use of such journals as a learning tool. Additionally, students engaged to different degrees with the learning journals so we cannot tell if briefer responses reflect a failure of learning or a failure to report on learning. Although this does not affect the outcome space, which reflects the qualitative nature of observed variation, we cannot determine the proportions of students in different categories which may be important for further research. Finally, as our study was conducted in one degree program in paramedicine in one university, extending the use and analysis of reflective journals to other sites and disciplines would test the broader relevance of the outcome space we have constructed.

## Conclusions

We have shown that reflective journals provide evidence for the extent to which students achieve complex learning outcomes in relation to understandings of and attitudes to EBP in paramedicine. Our phenomenographic analysis has revealed three key dimensions to these understandings and attitudes, suggesting that all three must be explicitly targeted for effective student learning. We have made some suggestions for how this could be achieved, emphasising the need to explicitly address epistemological development through social learning and metacognition. While our study does not address the apply and assess steps of EBP, it does suggest that barriers to integrating medical research and practice-derived knowledge are likely to impact on students’ capacity to implement these steps.

## Data Availability

The datasets generated and/or analysed during the current study are not publicly available due to the conditions set by the ethics approval process but are available from the corresponding author on reasonable request.

## Abbreviations

EBP: Evidence-based practice

## Author information

AW, SH and DH have backgrounds in educational research. AW and SH have additional backgrounds as scientific researchers. AH and A-MW are paramedicine educators.
